# Longitudinal Stability of Intellectual Functioning in Autism Spectrum Disorder: From Age 3 Through Mid-adulthood

**DOI:** 10.1007/s10803-021-05227-x

**Published:** 2021-10-22

**Authors:** Molly B. D. Prigge, Erin D. Bigler, Nicholas Lange, Jubel Morgan, Alyson Froehlich, Abigail Freeman, Kristina Kellett, Karen L. Kane, Carolyn K. King, June Taylor, Douglas C. Dean, Jace B. King, Jeff S. Anderson, Brandon A. Zielinski, Andrew L. Alexander, Janet E. Lainhart

**Affiliations:** 1 Department of Radiology and Imaging Sciences, Radiology Research, University of Utah, 729 Arapeen Drive, Salt Lake City, UT 84108 USA; 2 Department of Psychology and Neuroscience Center, Brigham Young University, Provo, UT USA; 3 Department of Neurology, University of Utah, Salt Lake City, UT USA; 4 Department of Psychiatry, University of Utah, Salt Lake City, UT USA; 5 Department of Neurology, University of California-Davis, Davis, CA USA; 6 Department of Psychiatry, Harvard School of Medicine, Boston, MA USA; 7 Department of Psychology, University of Utah, Salt Lake City, UT USA; 8 Waisman Center, University of Wisconsin-Madison, Madison, WI USA; 9 Department of Medicine, University of Wisconsin-Madison, Madison, WI USA; 10 Department of Pediatrics, University of Utah, Salt Lake City, UT USA; 11 Department of Pediatrics, University of Wisconsin-Madison, Madison, WI USA; 12 Department of Medical Physics, University of Wisconsin-Madison, Madison, WI USA; 13 Department of Psychiatry, University of Wisconsin-Madison, Madison, WI USA

**Keywords:** Autism spectrum disorder, ASD, Intelligence, Longitudinal study, Cognitive development, Stability

## Abstract

Intelligence (IQ) scores are used in educational and vocational planning for individuals with autism spectrum disorder (ASD) yet little is known about the stability of IQ throughout development. We examined longitudinal age-related IQ stability in 119 individuals with ASD (3–36 years of age at first visit) and 128 typically developing controls. Intelligence measures were collected over a 20-year period. In ASD, Full Scale (FSIQ) and Verbal (VIQ) Intelligence started lower in childhood and increased at a greater rate with age relative to the control group. By early adulthood, VIQ and working memory stabilized, whereas nonverbal and perceptual scores continued to change. Our results suggest that in individuals with ASD, IQ estimates may be dynamic in childhood and young adulthood.

**Supplementary Information:**

The online version contains supplementary material available at 10.1007/s10803-021-05227-x.

Measures of intelligence are important in the clinical evaluation of children and adults with Autism Spectrum Disorder (ASD) and in ASD research. Tests of intelligence, most commonly assessed as intelligence quotients (IQ) metrics or index scores, are typically obtained on all individuals who meet criteria for ASD because of the major implications for educational, vocational, and treatment planning and understanding vulnerability to distressing emotional states (Kraper et al., 2017; Mayes & Calhoun, 2003; Mayes et al., 2011; Pallathra et al., 2018; Solomon et al., 2018; Stewart et al., 2017; Tureck et al., 2014). Basic relations exist between level of intellectual functioning and social processing (Bishop-Fitzpatrick et al., 2017; Morrison et al., 2019); this relationship is a problematic area of cognitive processing and adaptive functioning in those with ASD (Kraper et al., 2017). Lower levels of intellectual functioning have been associated with higher levels of ASD symptom severity (Bishop et al., 2006; Charman et al., 2017; Mayes et al., 2011; Nordin & Gillberg, 1998) and are one of the strongest childhood predictors of diagnostic and functional outcome in adulthood (D. K. Anderson et al., 2014; Howlin et al., 2004, 2014; Magiati et al., 2014). Psychometrically, IQ correlates positively with essentially all other cognitive metrics, particularly language and memory (Prigge et al., 2013; Southwick et al., 2011). A general intelligence factor, “g”, is thought to mediate the interrelationships between all cognitive processes and IQ (Deary, 2012). Because a modest but positive correlation exists between IQ and regional brain volumes (Lange et al., 2010) and cortical thickness (Zielinski et al., 2014), psychometric computation of IQ scores has become an important matching and/or statistical control feature in neuroimaging research (Bigler, 2017). A better understanding of the IQ metric as assessed in individuals with ASD has broad implications for all facets of the condition.

Despite the importance of the IQ measure in the clinical care of individuals with ASD and in ASD research, very little is known about the stability of IQ longitudinally, especially during the transitions from childhood through adolescence and into adulthood (Martos-Perez et al., 2018). Cross-sectional studies have examined IQ in those with ASD from childhood to adulthood (Charman et al., 2017; Tillmann et al., 2019) but do not inform on individual changes. Longitudinal childhood studies show that some ASD participants have stable IQ scores (within ± 1 SD), yet many individuals have scores that increase or decrease over time (see Begovac et al., 2009 for a review; Solomon et al., 2018). The few studies that have tested ASD participants in childhood and repeated testing in young adulthood report an overall gain of 7 points (Simonoff et al., 2019) and significant variability (Bishop et al., 2015; Lord et al., 2015). Some studies have shown a decline in IQ in 23–35% of the participants, while 18–33% of the participants show an increase in IQ scores from childhood to adulthood (Farley et al., 2009; Howlin et al., 2014). To date, limitations of longitudinal ASD studies of IQ across the lifespan include lack of a control sample, estimates of IQ scores from adaptive functioning, and most importantly, a limited number of time points per individual (≤ 3) from which to infer longitudinal change or stability. Additional waves of data, of 3 time points or more, add to the measurement of individual trajectories and reliability of estimated change at the group level by statistical regression models (Willett et al., 1998).

The longitudinal Interdisciplinary Science to Learn about Autism (ISLA) project is an NIH-funded study of how clinical phenotypes and multimodal brain images change over time in ASD individuals. The goal of ISLA is to understand central tendencies, variation in brain development and maturation and the relationship to variation in clinical course and adult outcome. To date, the project has focused on the subgroup of individuals with autism whose cognitive ability (nonverbal IQ) is ≥ 70. Research has shown that adult outcome is poor to very poor in up to 60% of individuals in this subgroup (Howlin et al., 2013). In the ISLA study, participants were enrolled as young as 3 years of age and have been tracked to the current time frame; some participants have been followed and re-examined for more than 22 years with up to 7 time points of IQ data. Accordingly, we now describe the longitudinal trajectory and the stability of IQ scores in this ASD sample compared to age-matched, typically developing controls (TDC).

One challenge investigating IQ from childhood to adulthood is that its psychometric assessment is dependent on different standardized measurements that vary by age and language ability. When this investigation began, there was no universal IQ test that could be administered across the broad age-range of participants enrolled in the study, let alone over the next 20+ years of life. Furthermore, over a longitudinal study of this length, tests are updated and revised to reflect age and demographic normative adjustments due to drift over time. IQ for the youngest children was assessed by using either the Mullen Scales of Early Learning (Mullen, 1995) or the Differential Ability Scales (DAS; Elliott, 1990), both of which are established measures in the assessment of young children with ASD (Akshoomoff, 2006; Bishop et al., 2011). Once the participant was 5 or 6 years of age, intellectual assessment was performed with the DAS or one of the Wechsler measures [Wechsler Intelligence Scale for Children, WISC-III (Wechsler, 1991) or Wechsler Abbreviated Scale of Intelligence, WASI; (Wechsler, 1999)]. Once participants reached 16 years of age, a Wechsler-based test was used (Wechsler, 1997, 1999, 2008). Regardless of the test administered, all of the IQ measures employed in the ISLA project are highly interrelated and provide similar domain metrics that permit comparison across time (Flanagan & McDonough, 2018); using standardized IQ metrics from different tests becomes the practical solution when the identical test cannot be used in every instance, as is common practice in the real world (Bishop et al., 2011).

In the history of intellectual assessment, some core domains of functioning have emerged that encompass an overall or full-scale metric, assumed to characterize the level of aggregate intellectual performance. The Full Scale IQ (FSIQ) score, as a composite, is derived from verbal and non-verbal tasks and has age-adjusted norms with a mean of 100 (SD 15). Subdomains, while not all referred to as IQ scores, are nonetheless index scores with the same mean and SD, highly related to FSIQ but also representative of their own domain. Wechsler subset IQ or index scores have evolved over time and likewise influenced how other IQ tests categorize domains. These general domains include Verbal IQ (VIQ), Nonverbal IQ (NVIQ or Performance IQ), Verbal Comprehension Index (VCI), Perceptual Organization Index (POI), Working Memory Index (WMI) and Processing Speed Index (PSI) (Canivez et al., 2017; Wechsler, 1997). Within normative samples, IQ scores are assumed to stabilize by older adolescence and young adulthood, as shown by Salthouse (2016, 2019a, 2019b) and others (Hartshorne & Germine, 2015). Although different instruments for assessing IQ were used across our study period, the general domains used by the Wechsler approach can also be extracted from the Mullen or DAS (Flanagan & McDonough, 2018; Lezak et al., 2012). Thus, we have retained these classic intellectual and cognitive processing distinctions in terms of functional domains, although these non-Wechsler scales were administered to the youngest participants.

To our knowledge, ISLA is the first study to characterize intellectual functioning across more than 3 time points in most participants, spanning early childhood to mid-adulthood. We employed a straightforward method to investigate stability over time in ASD by plotting trajectories that link individual participants’ time points and then aggregating these data to generate a visual depiction of measured group changes across age. By their design, age-normed longitudinal standardized IQ scores are not expected to change over time at the general population level. Given the mixed age-related findings in studies to date, we tested the hypothesis that intelligence measures were stable over time in cognitively-able (nonverbal IQ ≥ 70) individuals with ASD and would follow similar trends to those observed during typical development. We present statistical analyses of age-related changes for the entire ASD sample and then the subset of participants age 18 and older to examine stability in IQ scores throughout adulthood. We also asked whether or not IQ scores remained within a level of expected variability given inherent fluctuations in cognitive performance that naturally occur over time and known test–retest variability and practice effects. These sources of extraneous variation can cause an IQ score to deviate beyond its inherent variability (see Hinton-Bayre, 2016; Maassen et al., 2009). Finally, we explored the longitudinal trajectories of IQ in the subgroup of ASD individuals with highly discrepant NVIQ and VIQs (≥ 15 points).

## Methods

### Participants and Study Design

ISLA uses an Accelerated Longitudinal Design, specifically, a mixed cross-sequential/

cohort-sequential strategy (Farrington, 1991). ASD participants were recruited from predominantly community and to a lesser extent clinical sources (namely parent support groups, youth groups, schools, and clinical social skills groups) into four age cohorts (3–6 years, 7–11 years, 12–18 years, and 19–39 years) and followed longitudinally; some participants had IQ scores from a prior study that began in 1997. For this report, we included all ASD and TDC participants with Intelligence measures collected from 1997–2018 (see Table 1). For comparison with other studies (D. K. Anderson et al., 2014; Howlin et al., 2013), only participants with at least one NVIQ score of 70 or above were included. Thus, of the 127 ASD participants tested, 5 participants were excluded because all available IQ scores were < 70, and 3 participants were excluded due to all testing being completed over the age of 41, and not overlapping in age with our TDC group. The resulting sample included 119 ASD participants (114 males, 5 females) and 128 TDC participants (122 males, 6 females). Ninety-nine (83%) of the ASD participants and 55 (42%) of the TDC participants were enrolled between 2002–2005; the remaining participants entered the study during subsequent years. All study procedures were approved by the University of Utah IRB.

**Table 1:** Participant Characteristics

	ASD n = 119		TDC n = 128		
	Mean (SD)	Range	Mean (SD)	Range	t-value
Initial Visit: age in years	14.2 (8.1)	3.1–36.8	20.8 (8.5)	3.6–39.7	6.2 (p < .001)
Initial Visit: % sample					
Early Childhood (3–6 years)	24%		6%		
Middle Childhood (7–11 years)	24%		13%		
Late Childhood (12–18 years)	26%		15%		
Adulthood (19+ years)	26%		66%		
All Visits: age in years	19.6 (9.0)	3.1–46.4	20.1 (8.1)	3.6–39.7	0.7 (ns)
% Sample with IQ Discrepancy at Time 1					
NVIQ > VIQ ≥ 15	24%		5%		
VIQ > NVIQ ≥ 15	13%		14%		
Parental Education (yrs)					
Mother’s	15.6 (2.0)	12–21	15.7 (2.4)	12–21	0.1 (ns)
Father’s	16.7 (2.6)	12–24	16.8 (2.6)	12–23	0.2 (ns)
ADI-R Social	19.5 (5.6)	8–30			
ADI-R Communication	15.4 (4.3)	7–25			
ADI-R RRB	7.0 (2.3)	3–12			
ADOS-2 Total CSS	8.2 (1.6)	2–10			
ADOS-2 SA CSS	8.1 (1.5)	2–10			
ADOS-2 RRB CSS	7.6 (1.9)	1–10			

Two of the ASD participants had ADOS Total scores below the ASD cutoff of 8 at time of study entry, resulting in low CSS scores. Review of early clinical data combined with ADI-R confirmed lifetime diagnosis of ASD

*ADI* Autism Diagnostic Interview-Revised; *ADOS* Autism Diagnostic Observation Scale; *CSS* ADOS-2 calibrated severity score; *SA* Social Affect; *RRB* Restricted and Repetitive Behaviors

ASD was diagnosed using DSM-IV (American Psychiatric Association, 1994) and ICD-10 criteria, and was based on assessment with the Autism Diagnostic Interview-Revised (ADI-R; (Lord et al., 1994), the Autism Diagnostic Observation Schedule (ADOS; (Lord et al., 1999) or ADOS-2 (Lord et al., 2012), and record review. Collaborative Programs of Excellence in Autism (CPEA) criteria (Lainhart et al., 2006) were used for an ADI-R classification of ASD. ADOS scores were converted to ADOS-2 algorithm scores and comparison scores on all ASD participants (Hus & Lord, 2014; Hus et al., 2014). ADI-Rs and ADOS/ADOS-2 s were administered by research-reliable clinicians and PhD-level research staff. ASD participants met criteria for a classification of ASD on both the ADI-R and ADOS-2 at study entry, with the exception of 2 older participants who met ADI-R criteria and whose records were consistent with a lifetime diagnosis of ASD, but their current ADOS scores were subthreshold. ASD was idiopathic in all cases; known medical causes of ASD were excluded (Lange et al., 2015).

We compared longitudinal intelligence measures in our ASD group to measures in our TDC group. The TDC group helped account for possible changes in intelligence scores over time related to (a) different tests used, particularly in childhood versus adulthood (see below), (b) secular changes, (c) intelligence score inflation, and (d) site-specific effects. All TDC participants were assessed with the ADOS/ADOS-2 to rule out ASD, they had to score within 1 SD of the mean on standardized tests of IQ, language, memory, and adaptive function, and they had no history of developmental, neurological, or psychiatric disorder. Beginning in 2007, we tried to enrich the non-ASD sample for children with IQs between 70–84, but only one of the many children in this IQ range who we assessed was otherwise “typically” developing and thus included in this study.

### Intelligence Tests

At each timepoint, intelligence testing was obtained as part of a larger neuropsychological battery. At Timepoint 1, IQ tests for child participants (≤ 16 years) were based on age and language ability (Mullen vs DAS). Scores obtained from testing prior to Timepoint 1 and all subsequent timepoints included only Wechsler based tests. Table 2 summarizes the tests used to measure intelligence across the cohorts and within individual participants. These measures provide repeated estimates of FSIQ, NVIQ, and VIQ, VCI, POI, WMI, and PSI. To examine changes in general intelligence measures over time, FSIQ obtained from the Wechsler tests was combined in the same analysis with the General Conceptual Ability Score (GCA) of the DAS and Early Learning Composite from the Mullen. The DAS Verbal Cluster was the best estimate of Wechsler VIQ. The Special Nonverbal Composite of the DAS (School and Lower Preschool versions) and Nonverbal Cluster from the DAS Upper Preschool forms were used as NVIQ estimates. For different Wechsler versions, the WAIS-IV (Wechsler, 2008) VCI was used as the WAIS-III/WISC-III VIQ and VCI equivalents, WAIS-IV Perceptual Reasoning Index as WAIS-III/WISC-III NVIQ and POI estimates, and WISC-III Following Directions Index as WAIS-III/WAIS-IV WMI equivalent. A master table summarizing intelligence measures and index scores broken down by test version is provided as an online Supplementary Table. All intelligence tests were administered by clinical research scientists and graduate-student research staff, trained and supervised by senior clinical research neuropsychologists.

**Table 2:** Number of Intelligence Tests Administered

	ASD group	TDC group
	436 tests/n = 119	222 tests/n = 128
	# tests/# participants	# tests/# participants
Mullen	1/1	0
DAS-Preschool	17/17	3/3
DAS-School Age	42/42	26/26
WISC-III	36/32	10/10
WASI	175/84	95/49
WAIS-III	134/100	88/86
WAIS-IV	31/31	0

*Mullen* Mullen Scales of Early Learning (Mullen, 1995), *DAS* Differential Ability Scales (Elliott, 1990), *WISC* Wechsler Intelligence Scale for Children (Wechsler, 1991), *WASI* Wechsler Abbreviated Scale of Intelligence (Wechsler, 1999), *WAIS* Wechsler Adult Intelligence Scale (Wechsler, 1997, 2008)

As mentioned in the Introduction, the challenge of the current investigation in obtaining typical and reliable change indices is that the same intellectual assessment instrument was not used across all participants and time points. To address this, we sought a practical solution by using general clinical decision rules where practice guidelines assume that repeated IQ scores within ± 0.5 SD are within a cone of normal variability, representing fluctuations of no particular clinical importance (Lezak et al., 2012). We applied this rule to the current dataset and examined the frequency of IQ scores that deviated beyond ± 0.5 SD, using the initial IQ score as baseline or middle IQ score as an “anchor” in those with more than 2 scores. A second approach used the actual 95% confidence interval (CI) derived from the Wechsler-based IQ scores. Because all IQ measures used were based on a mean of 100 and a SD of 15, using this approach permitted comparison of the IQ metric regardless of which test procedure was used in obtaining the IQ score.

### Statistical Analysis

Linear mixed effect models (Laird & Ware, 1982; Lange & Laird, 1989) were employed to compare longitudinal changes in IQ scores over time in the ASD group to those in the TDC group. This strategy allowed for different numbers of testing occasions across participants, and it allowed inclusion of individual random intercepts and slopes for all participants. Each intelligence measure was modeled with a fixed component accounting for the linear effects of age, group, an age*group interaction, and for a quadratic effect of age and its interaction with group. Best fitting models were identified by the Akaike Information Criterion (Akaike, 1974). For each analysis, age was mean centered (combined ASD + TDC sample) and a Bonferroni correction was applied (significant < 0.05/7 = 0.007) to account for the seven intelligence indices being analyzed. Longitudinal changes in NVIQ-VIQ discrepancy scores were also examined using mixed models. We then examined the stability of intelligence scores within individuals with ASD by calculating intraclass correlation coefficients (ICCs) and reporting the percentage of participants with repeated testing scores within ± 0.5 SD and 95% Confidence Intervals (CIs) of established testing norms. All mixed effects models were run using the nlme package in R (Pinheiro et al., 2017; R Core Team, 2017), ICCs calculated using the R package ICC (Wolak et al., 2012).

## Results

### Descriptive Statistics

Mean age at the time of first IQ measurement was lower in the ASD group compared to the TDC group, but mean participant age across all tests collected did not differ between groups [see Table 1 and Supplementary Fig. 1 for age distribution of participants into age cohorts of early childhood (3–6 years), middle childhood (7–11 years), adolescence (12–18 years) and adulthood (19+ years)]. ASD and TDC groups were well-matched on parental education.

Most (77%) of the 99 ASD participants and 56% of the 55 TDC participants in the original cohort recruited into the longitudinal study between 2002–2005 had ≥ 3 time points of IQ data and 69% ASD and 30% TDC with ≥ 4 time points. Participants recruited after 2005 had fewer time points of data, resulting in 68% of the total ASD sample and 24% of the TDC sample with ≥ 3 IQ data points and 58% of ASD and 13% TDC with ≥ 4 data points. The lower rate of repeated IQ measures in the TDC sample decreased the reliability of the longitudinal TDC IQ trajectories at the group level compared to the ASD trajectories (Willett et al., 1998). Nevertheless, the TDC group’s IQ trajectories generally show relative stability of mean scores, which is to be expected given that IQ tests are normed for all ages. To ensure that trajectories of the ASD IQ curves were not influenced by more severe cases having fewer IQ scores, we compared ASD participants with and without ≥ 4 time points of data; the 2 groups did not differ on mean age at first test (t = 1.4), first FSIQ score (t = 0.58), parental education (mother t = 2.1, father t = 1.8), or ADOS total calibrated severity score (t = 1.6). Due to the wide age range of participants, we also examined whether or not ADI Algorithm scores differed in the participants who were children (3–17 years) vs adults (18 years+) at study entry. We found similar Communication (t = 0.01) and Restricted, Repetitive Behavior scores (t = 0.3) yet higher Social Interaction scores in the adult group (mean = 22.6, child mean = 18.9; t = 2.6, p = 0.02). None of our ASD participants became untestable over time.

### Longitudinal Trajectories of IQ and Index Scores in ASD

**Fig. 1:**
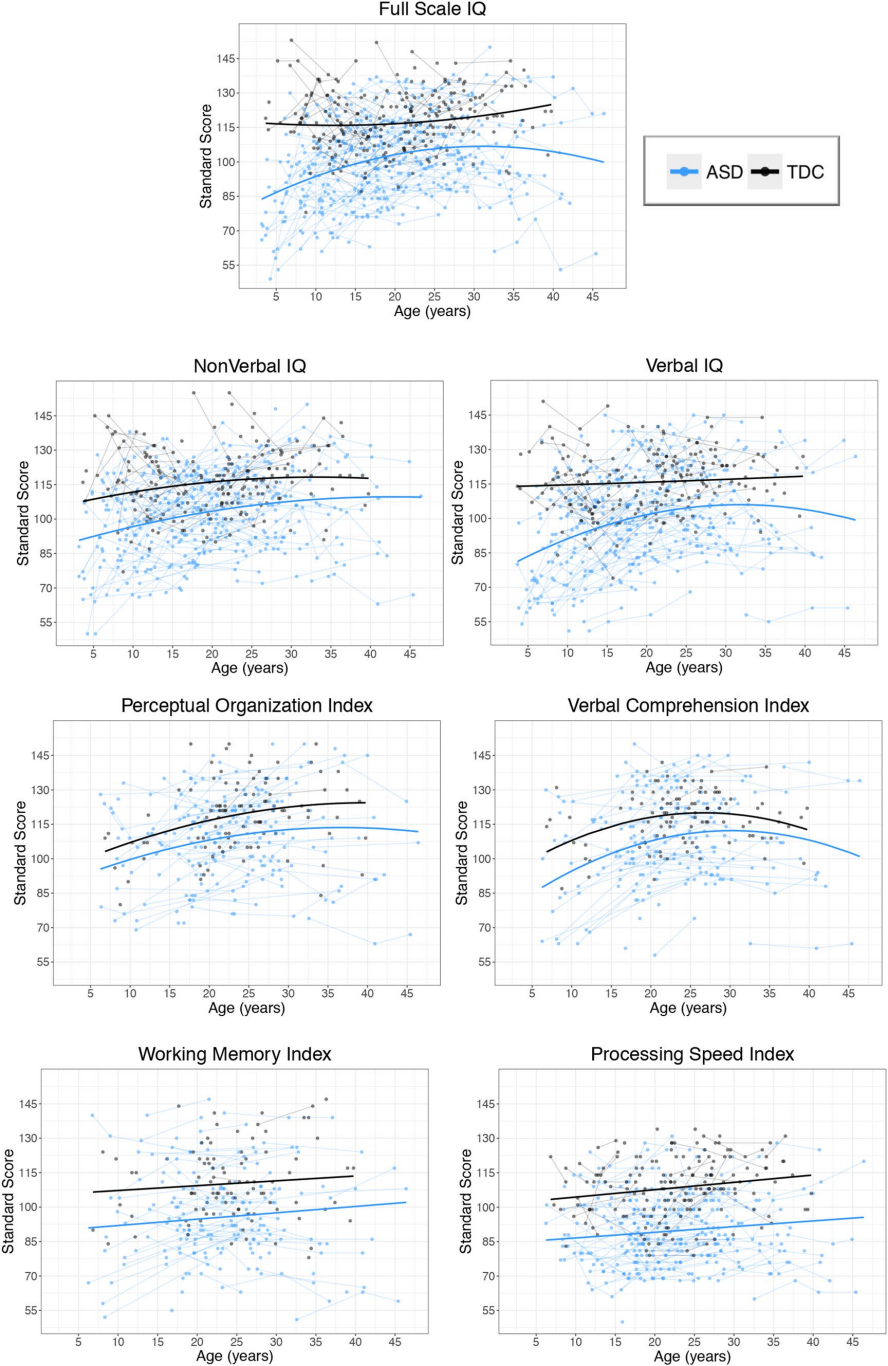
Longitudinal changes in intelligence scores over time in ASD and TDC

Figure 1 depicts the longitudinal IQ scores and age-related changes in both the ASD and TDC groups. FSIQ, VIQ, NVIQ scores were available for all ages and POI, VCI, WMI and PSI were available for participants 6 years and up. Table 3 contains the parameter estimates from the fitted mixed effects models examining age-related changes in ASD and group by age interactions. Visual inspection of Fig. 1 across the different IQ metrics examined, along with their quantitative analyses, shows the expected lower mean intelligence quotients and also lower index scores in the ASD group compared to the TDC group. After controlling for multiple comparisons, we found that all scores increased significantly with age in the ASD group except for WMI and PSI. FSIQ and VIQ started lower in the youngest cohorts of ASD participants and increased at a greater-than-typical rate with age. Our PSI findings replicate and expand on the subgroup of participants with up to 3 time points described previously (Travers et al., 2014).

**Table 3:** Parameter Estimates from the Mixed Effects Models

IQ scores: Mullen, DAS, WISC-III, WAIS-III, WAIS-IV, WASI						
	ASD Intercept	Group	Age	Age^2^	Group x Age	Group x Age^2^
FSIQ	103.0	13.5***	0.67***	− 0.03***	− 0.49***	0.04***
VIQ	101.6	14.1***	0.74***	− 0.03***	− 0.61***	0.03
NVIQ	103.3	12.7***	0.55***	− 0.01	− 0.23	

Index scores: WISC-III, WAIS-III, WAIS-IV						
	ASD Intercept	Group	Age	Age^2^	Group x Age	Group x Age^2^
VCI	110.1	9.4***	0.60***	− 0.04***	− 0.30	
POI	110.0	8.9***	0.53***	− 0.02	0.12	
WMI	95.8	14.3***	0.27		− 0.06	
PSI	89.7	18.7***	0.25		0.07	

ASD group is the reference group. ***p < 0.007 (significant at p < 0.05 after Bonferroni correction)

*FSIQ* Full Scale Intelligence Quotient, *VIQ* Verbal Intelligence Quotient, *NVIQ* Performance Intelligence Quotient, *VCI* Verbal Comprehension Index, *POI* Perceptual Organization Index, *WMI* Working Memory Index, *PSI* Processing Speed Index

Within-individual trajectories of the TDC participants supports the well-known increased instability of high-IQ scores during childhood and regression toward the mean, in which extreme values observed on the first measurement of a phenomenon “regress” toward the population mean on subsequent measurements (Galton, 1889; Rinaldi & Karmiloff-Smith, 2017). The large differences in the estimated values of the ASD and TDC longitudinal trajectories at the youngest and oldest ages are based on smaller amounts of IQ data and are best interpreted with caution.

#### Adult Group Only

To examine whether or not age-related changes were driven only by the younger ASD participants or persisted within the adult ages, we reran the models summarized in Table 3 but only included participants age 18 years and older at test date (ASD n = 93, 227 test visits; TDC n = 98, 132 test visits; mean age in years ASD = 26.6 years, TDC = 25.6, t = 1.5 p = ns). All versions of the Wechsler tests WAIS-III, WAIS-IV, or WASI were present in the adult subgroup. In our adult ASD sample, age was significantly related to increasing standard scores of FSIQ, NVIQ, POI and PSI (Age effects: FSIQ ß = 0.80 p < 0.001, NVIQ ß = 0.85 p < 0.001, POI ß = 0.72 p < 0.001, PSI ß = 0.49 p < 0.006). In contrast to the whole group analysis, we no longer found significant age effects for VIQ and VCI and continued to find no age effect for WMI (VIQ ß = 0.17 p = ns, VCI ß = 0.28 p = ns, WMI ß = 0.06 p = ns). There were no longer significant group*age interaction effects for FSIQ and VIQ (FIQ ß = 0.006, VIQ ß = − 0.19). These findings suggest stable VIQ and WMI scores during adulthood in ASD. Adult trajectories are presented in Supplementary Fig. 2.

#### Longitudinal IQ Trajectories in ASD at the Level of Individuals

**Fig. 2:**
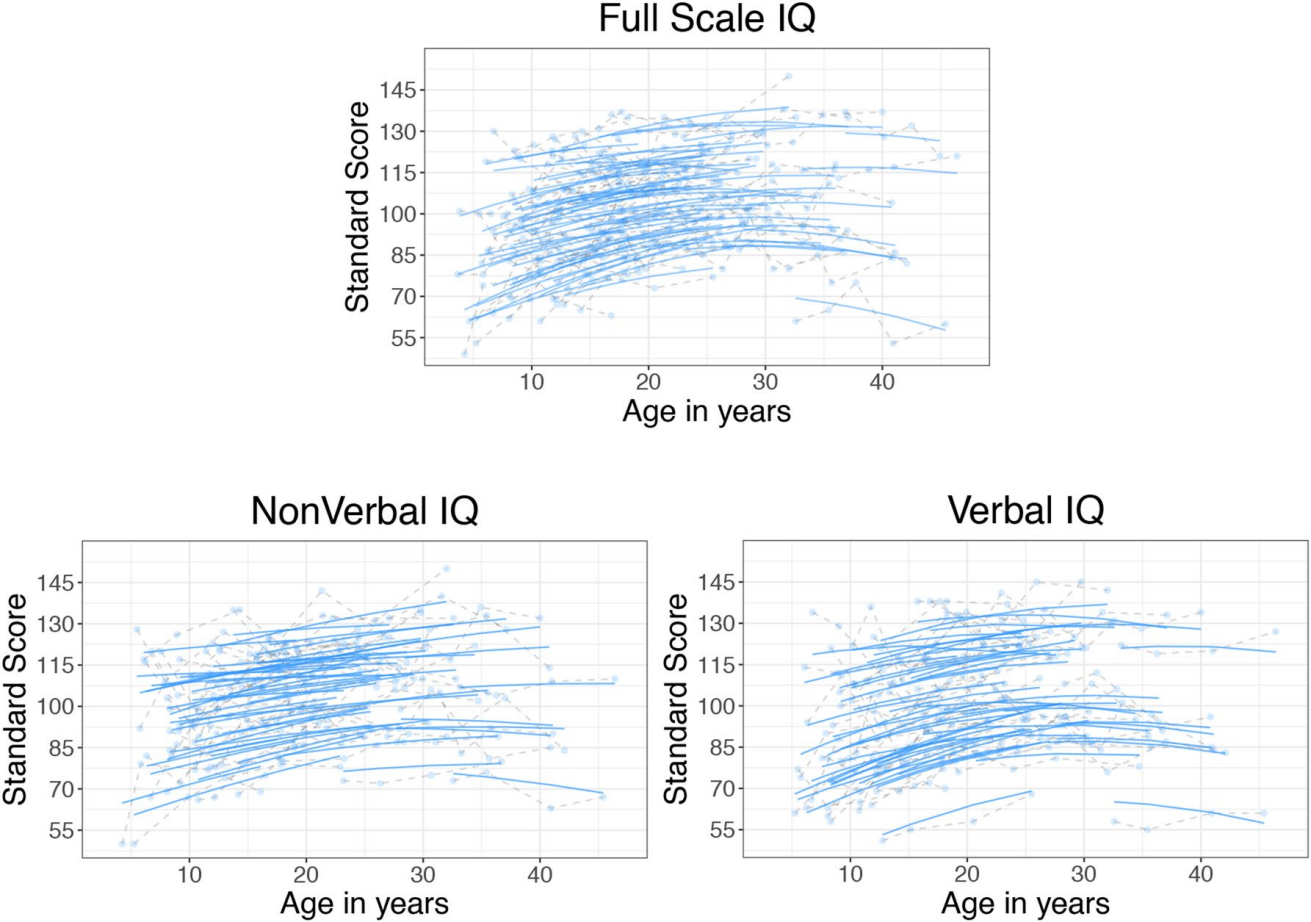
Individual predicted change estimates for ASD participants with 4+ time points

Figure 2 displays the estimated change over time for FSIQ, NVIQ and VIQ in those ASD participants with ≥ 4 IQ datapoints. Individual curves were predicted from the contributions of the fixed and random effects from the mixed effects models. Variability between individuals is evident, with some participants showing scores that increase over time, some are stable over time, and others slightly decline. Examination of within-individual trajectories of the ASD participants did not show evidence of a subgroup with sustained significant atypical age-dependent decline of ≥ 15 points across developmental periods or in adulthood. We have provided a figure showing the relationship between random intercepts and slopes in those participants with both childhood and adulthood scores as Supplementary Fig. 3.

### Longitudinal Changes in NVIQ > VIQ Discrepancy in ASD at the Subgroup Level

**Fig. 3:**
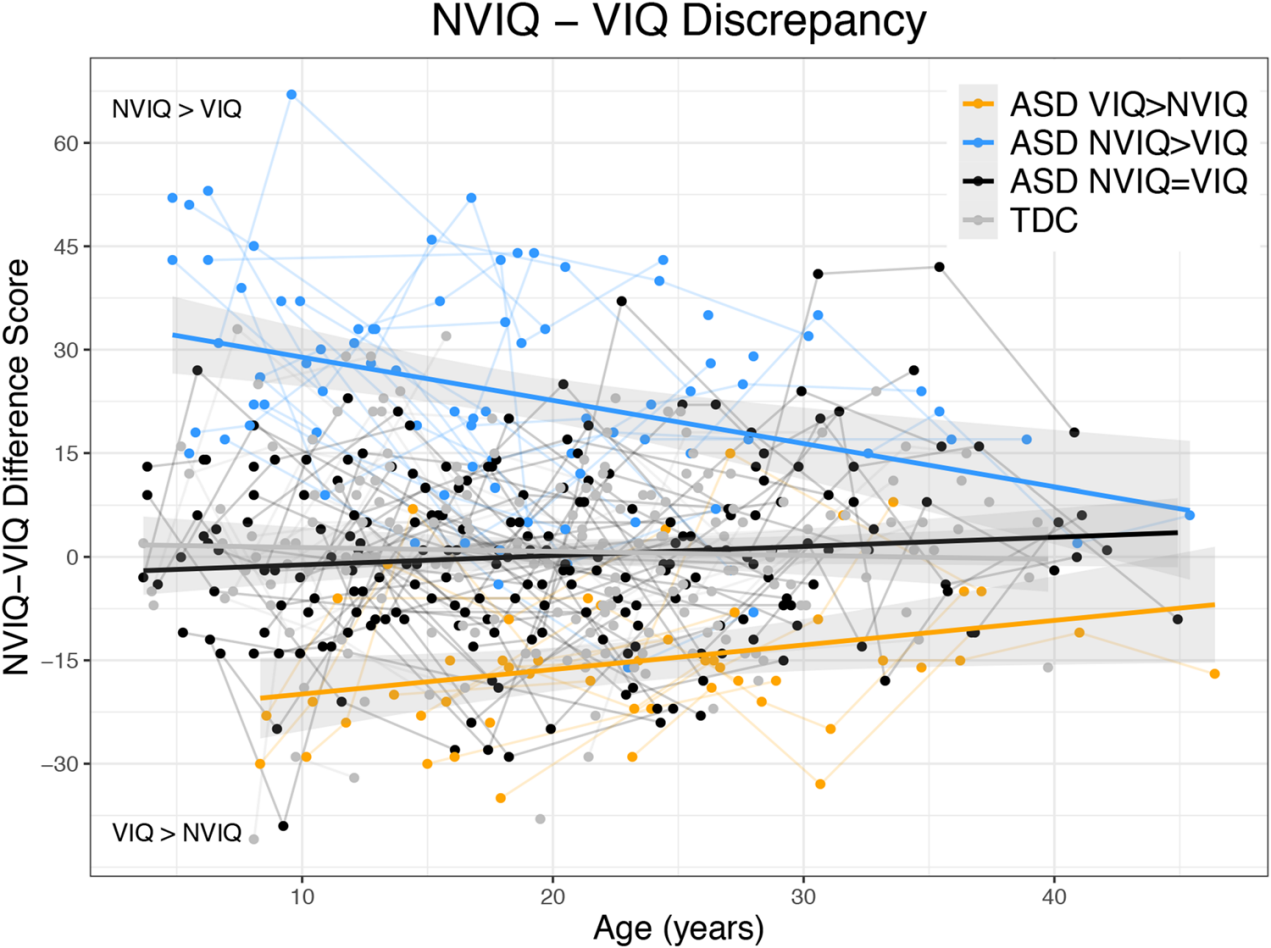
NVIQ-VIQ discrepancy score changes in ASD subgroups

NVIQ > VIQ discrepancy in ASD is associated with large head size, increased impairment in social functioning, and a decreased rate of loss-of-function mutations and copy number variants in ASD-associated gene loci compared to ASD individuals without discrepant IQs (Bishop et al., 2017; Deutsch & Joseph, 2003; Joseph et al., 2002). We explored the longitudinal trajectory of NVIQ > VIQ discrepancy in this hypothesized neurobiological cognitive subtype of ASD. Twenty-eight of the ASD participants (24%) had NVIQ 15 or more points higher than VIQ when first tested and 16 participants (13%) had VIQ 15 or more points higher than NVIQ. As evident in Fig. 3, the NVIQ > VIQ subgroup of participants showed a significant decrease in NVIQ-VIQ discrepancy score with age that was not found in the rest of the ASD group (group*age interaction ß = 0.75, p < 0.0002) or TDC participants (group*age interaction ß = 0.7, p = 0.001). A post-hoc analysis showed that the decrease in magnitude of the NVIQ > VIQ discrepancy with age in this subgroup was driven by an increase in VIQ with age above that found in the non-discrepant IQ ASD group. Supplementary Fig. 4 shows the versions of IQ tests administered over time within the ASD discrepant subgroups.

### Stability of Within-Individual IQ Scores Over Time

We examined stability of IQ and index scores over time by calculating ICCs for the ASD and TDC participants (all ages, all IQ tests) with repeated testing (see Table 4). In the ASD group, ICCs ranged from 0.75 for NVIQ to 0.85 for WMI. In the TDC group, ICCs ranged from 0.58 for NVIQ to 0.74 for FSIQ (see ICC column in Table 4). Although ICCs were in general higher in ASD, there were also a greater number of participants with repeated tests available for analysis.

**Table 4:** The Number of Participants with Repeated Intelligence Scores

	ASD							TDC					
	2*	3	4	5	6	7	ICC	2*	3	4	5	6	ICC
FSIQ	16	12	17	36	14	2	0.80	15	15	15	1		0.74
VIQ	22	18	34	14	5		0.78	19	18	2			0.76
NVIQ	23	20	34	14	5		0.75	19	18	2			0.58
VCI	33	21	5	1			0.78	2					
POI	33	21	5	1			0.78	2					
WMI	34	21	2				0.85	2					
PSI	23	32	23	6			0.81	27	8				0.74

*For example, FSIQ was collected 2 times on 16 ASD participants and 15 TDC participants.

*ICC* Intraclass Correlation Coefficient, *FSIQ* Full Scale Intelligence Quotient, *VIQ* Verbal Intelligence Quotient, *NVIQ* Performance Intelligence Quotient, *VCI* Verbal Comprehension Index, *POI* Perceptual Organization Index, *WMI* Working Memory Index, *PSI* Processing Speed Index

To reduce extraneous variability in test score that may be due to instrument version or change over developmental period (child to adolescent to adult) we then examined stability of IQ scores over time in our ASD participants with more than one full adult Wechsler testing session (WAIS-III or WAIS-IV, participants age 16+ years). Out of the 50 ASD participants with repeated full Wechsler testing, 2 tests were available from 37 participants, 3 tests from 12 participants and 4 tests from one participant, with an average inter-session interval of 7.6 years (range 1.8–15.1 years). Only 2 TDC participants had repeated full Wechsler testing and are not reported. ICCs for the ASD scores increased from the full sample reported in Table 4: FSIQ = 0.91, NVIQ = 0.84, VIQ = 0.92, VCI = 0.90, POI = 0.86, WMI = 0.92. Similar to the adult mixed model results discussed previously, higher ICCs and more stable IQ metrics were found in the verbal and working memory domain. Repeated PSI scores from the WAIS-III or WAIS-IV were available for 79 ASD and 25 TDC participants. Two scores were available on 26 ASD and 18 TDC, 3 scores on 34 ASD and 7 TDC, 4 scores on 16 ASD and 5 scores on 3 ASD (inter-session interval: ASD range 1.0–8.8 years, average 4.7 years; TDC range 1.8–8.2 years, average 3.9 years). The PSI ICC was 0.84 in ASD and 0.79 in TDC.

Test stability was also measured by confidence intervals (CIs). The 95% CIs provided in Appendix A of the WAIS-III manual were calculated around the first score in those with only 2 tests or middle score in those with more than 2 tests. Both WAIS-III and WAIS-IV tests were combined for this analysis. Table 5 is a display of the percentage of participants with repeated test scores falling within these CIs. For the participants age 16+ years with repeated PSI scores (n = 79 ASD, n = 25 TDC), the percentage of participants within certain CIs was similar for the ASD and TDC groups.

**Table 5:** Percentage of ASD Participants Age 16+ with Repeated Full Wechsler Test Scores that Fell within Certain CIs

	One SD CI (± 7.5 points)		WAIS-III 95% CI	
	All scores within CI	No scores within CI	All scores within CI	No scores within CI
FSIQ	63%	27%	33%	59%
PIQ	51%	35%	45%	39%
VIQ	59%	33%	45%	47%
VCI	57%	33%	47%	45%
POI	59%	37%	47%	43%
WMI	63%	33%	55%	39%
PSI	50%	22%	65%	14%

FSIQ, PIQ, VIQ, VCI, POI, WMI: N = 50 ASD; PSI: N = 79 ASD. Confidence Intervals (CIs) were calculated around the first test score in those with only 2 scores or middle score in those with 3+ tests available. WAIS-III CIs were used for those participants with WAIS-IV scores

## Discussion

Our cognitively-able ASD sample demonstrated distinct differences in comparison to our age- and SES-matched TDC sample. First, the longitudinal trajectory of IQ differed in the ASD group compared to the TDC group. Mean IQ started lower than typical in early childhood, increased during childhood and adolescence, and continued to increase in young adulthood. Second, the trajectories of WMI and PSI were flat during childhood and adolescence in the ASD group and only PSI showed some increase in young adulthood. Third, in the subgroup of ASD participants with NVIQ significantly greater than VIQ when first tested, the IQ discrepancy decreased with age. Fourth, despite the different IQ tests used across age and developmental periods in this study, similar to repeated testing to estimate intellectual functioning from childhood into adulthood for clinical, educational, and vocational planning purposes, the test–retest stability over long periods was overall good, more so for VIQ than NVIQ. However, even after 16 years of age, we found significant within-individual variability: over 50% of ASD participants with repeated IQ testing had at least one IQ falling outside the 95% confidence interval. Fifth, when longitudinal individual IQ trajectories were plotted for participants with 4 or more time points of IQ measures, we did not observe a significant, sustained, age-related decrease in IQ from childhood to adolescence to adulthood or in adulthood in any ASD participants.

### Longitudinal Trajectory of IQ and Index Scores in ASD at the Group Level

The longitudinal trajectory of standardized IQ and some Index Scores at the group level in ASD is compatible with brain plasticity during childhood and even into young adulthood. ASD participants showed significantly lower IQ levels compared to those of TDC participants; this discrepancy was most evident in the younger ASD participants. As seen in Fig. 1, a substantial number of ASD participants had IQ scores below 85, which is one SD below the mean, whereas only a few TDC participants had IQ scores at or below that level. Because statistical analyses demonstrated that FSIQ and VIQ increased at a greater rate with age in the ASD group than TDC participants, it may be that at the earliest age, the IQ discrepancies between ASD and TDC are at their greatest. Because some component of communication impairment is present for the diagnosis of ASD, this impairment may be more associated with verbal processes. This in turn is reflected in the lowest VIQ scores for the youngest ASD participants with the steepest increases when compared to NVIQ. It has long been discussed that deficits in VIQ may be more likely to accompany the diagnosis of ASD than NVIQ, although this distinction is not always observed (Charman et al., 2011).

Recently, Solomon et al. (2018) examined intellectual development in an ASD sample of 102 children from 2 to 8 years of age. From initial study recruitment to age 8, over half of the children displayed improved intellectual scores, whereas approximately one-quarter showed decline. The Solomon study only examined ASD participants, so no similar comparison with TDC could be made. Figure 1 and our mixed model findings show that age-related increases in FSIQ and VIQ were driven by our child and adolescent participants. This finding is consistent with a recent population-based study of ASD children from 10–12 to 23 years of age (Simonoff et al., 2019). In Fig. 1, while overall intellectual functioning and the various index scores show increases with age, the curves of the ASD and TDC groups never intersect for FSIQ, VIQ and NVIQ, suggesting that reduced intellectual functioning at the group level for those with ASD is a common observation.

### Longitudinal Trajectory of IQ in ASD at the Individual Level

The longitudinal trajectories of IQ at the level of individuals with ASD in our sample suggest that despite between-individual variability, IQ does not significantly decline with age in a sustained manner from childhood to mid-adulthood in otherwise intellectually able individuals with autism. The finding is consistent with what is observed clinically in the rare case of an individual with ASD with a true sustained significant decrease in IQ over time: rather than being a feature of medically uncomplicated ASD, the individual may have developed a seizure disorder, a severe psychiatric disorder such as severe depression, or another medical condition that impacts cognition.

A substantial number of individuals with ASD in our study had high IQ scores that completely overlapped with TDC participants who achieved the highest possible levels of the IQ tests. Preliminary evidence on a small sub-sample of individuals with ASD who had high IQs in young adulthood suggests that higher VIQ during early childhood predicts higher adult functional outcome (Lord et al., 2020; Pickles et al., 2020). Nevertheless, what Kraper et al. (2017) wrote continues to hold true: “For individuals with autism spectrum disorder (ASD), long-term outcomes have been troubling, and intact IQ has not been shown to be protective” (p. 3007). While average to above average intellectual ability is a highly valued attribute, the neural deficits that underlie social cognition and emotional control may be far more important for actual adaptive functioning than IQ (see Mazefsky et al., 2014). There may be unique relations between brain structure and IQ yet to be discovered that affect relevance for social-emotional function.

### Implication of Stability and Instability of IQ in ASD

Variability in test scores represents a major observational finding of the current study. Although test scores across time within each domain were significantly and positively correlated, there was still considerable variability between time points. Individual test points can be identified and it is evident that some individuals in both groups exhibited variability in both directions (increasing or decreasing performance) on IQ testing over time. In our examination of individual stability, generally only half of all scores fell within the boundaries of either a 95% CI or ± 0.5 SD. This suggests that an IQ estimate for an individual obtained at an early age may significantly differ from IQ at a later age. In our sample, we found that by early adulthood, verbal and working memory scores exhibit the greatest stability. In a practical and clinical sense, this suggests that a single VIQ test in adulthood may be sufficient to estimate function throughout adulthood, but the same is not true in childhood, particularly early childhood. This conclusion is consistent with the literature survey findings of Begovac et al. (2009) that by late childhood, IQ metrics are relatively stable. In contrast, nonverbal and perceptual organization scores continued to increase during adulthood in our ASD sample. Whether these findings represent practice effects or brain and cognitive maturation attributing to improved performance over time remains an important question. In healthy adults, memory scores can increase or decrease with repeated testing but 2 or more scores beyond a 90% CI is not typical (Brooks et al., 2016). Our data suggest that, like other traits such as head circumference, intellectual functioning in ASD may be best understood by the longitudinal trajectory of multiple IQ points over time rather than any single IQ score at any one point in time. This finding has both research and clinical implications.

### Limitations and Future Directions

The biggest limitation of our study, due to resources, was the focus on individuals with ASD who had IQs ≥ 70, were verbally able, and male. We therefore do not know if our findings apply to females or to individuals with ASD who are also intellectually and verbally disabled. It is our hope that what we learn from our sample may eventually be relevant to these other very important groups. Our study findings are also limited in that our early childhood sample had a TDC group that was smaller and not IQ matched to the ASD group. This could have impacted our ability to estimate TDC trajectories during childhood and interpretation of group differences throughout the age range studied. Another limitation is the absence of detailed social, economic, and environmental history that may impact cognitive development and test performance.

There is also a major, but at the time unavoidable, limitation of the current study given that specific IQ and Index Scores varied between time points and were not uniformly administered. While a limitation, this is clinically realistic for professionals who evaluate and provide care for individuals with ASD across the lifespan and educators working with ASD students. It is possible that the different IQ metrics used to assess the youngest participants contributed to higher variability at earlier ages and potentially the lowest VIQ scores. For older individuals for whom only Wechsler versions were used, there is higher positive intercorrelation amongst versions than across task correlations with non-Wechsler intellectual assessment methods, although all measures are positively and significantly correlated (Flanagan & McDonough, 2018). Possibly the greater uniformity of the Wechsler-based procedures may have added to the stability of test scores observed in older childhood and adolescence through adulthood. The inclusion of a TDC group controls, in-part, for IQ test variation across the years of the study, because those TDC participants were taking the same tests at approximately the same times and intervals, especially when the participants were younger versus when they were older. Nonetheless, given known potential for test score inflation effects with IQ measures, the so-called “Flynn” effect (Kanaya & Ceci, 2012, 2018), and given possible secular trends in IQ over time (Bratsberg & Rogeberg, 2018), we have not likely been able to consider or statistically control for all variables that may have affected IQ in our longitudinal sample. However, for clinicians who examine, track and follow individuals with ASD, the findings presented herein do address some practical issues about the stability of IQ metrics from childhood into adulthood.

## Conclusion

This ISLA Utah Longitudinal Autism Study is the first to characterize intellectual functioning across multiple time points spanning early childhood to mid-adulthood. This investigation also has a research arm devoted to advanced MRI imaging analyses in ASD including diffusion tensor imaging, resting state functional connectivity mapping and standard structural neuroimaging (Alexander et al., 2007; J. S. Anderson et al., 2011; King et al., 2018; Lange et al., 2015; McLaughlin et al., 2018; Travers et al., 2015; Zielinski et al., 2012; Zielinski et al., 2014). This imaging is designed to probe the neural correlates of intellectual functioning in ASD and if they differ over time. Accordingly, the next step will be to examine these intellectual variables in ASD and TDC participants from a neurodevelopmental and neuroanatomical maturation perspective. Interesting findings relating important developmental parameters using brain imaging methods have begun with cross-sectional studies (Grydeland et al., 2019; Maier et al., 2018); however, the Utah sample will be particularly unique to further tease out the various brain-behavior relations based on a within subject longitudinal investigation to further our understanding between brain structure and function, IQ and autism (Hampshire et al., 2012). Additionally, the ISLA study has also obtained other neurocognitive, motor and neurobehavioral measures on this same ASD and TDC sample (Duffield et al., 2013; Green et al., 2016; Jantz et al., 2015; Travers et al., 2017). As mentioned in the Introduction, the “g” factor relations and their stability and/or variability between IQ and these other measures, while assumed to be similar for TDC, is unknown in ASD. Answers to these questions will be forthcoming.

## Supplementary Information

Below is the link to the electronic supplementary material.

Supplementary file1 (DOCX 321 kb)

Supplementary file2 (XLSX 14 kb)
