# Supplementary material for: Longitudinal Stability of Intellectual Functioning in Autism Spectrum Disorder: From Age 3 Through Mid-adulthood
Source: J Autism Dev Disord. 2021 Oct 22;52(10):4490–504. doi: 10.1007/s10803-021-05227-x (PMC9090201; doi:10.1007/s10803-021-05227-x)
Supplement: Supplementary file 1 — Supplementary file1 (DOCX 321 kb) [file 10803_2021_5227_MOESM1_ESM.docx]

Supplementary Material.

Supplementary Figure 1: Age of participants at testing

Supplementary Figure 2. IQ score changes in the adult only sample

Supplementary Figure 3. Relationship between Random Intercepts and Slopes

**Our best fitting models for FSIQ, NVIQ and VIQ were rerun with age re-centered at 7 years, allowing us to extract the IQ estimate during early to mid-childhood. Below are the random intercepts and slopes for our participants with study visits during both early or middle childhood and again in adulthood (ASD n=30). These plots show that the participants with negative random intercepts, or lower than expected scores during childhood, have more positive random slopes, or greater IQ score change over time.

Supplementary Figure 4. Longitudinal change in NVIQ-VIQ Discrepancy and IQ test version.

This figure shows longitudinal NVIQ-VIQ discrepancy scores in the ASD participants with 3+ IQ tests. The different IQ tests are represented by different colors, showing how individuals change over time and the different IQ versions they received.
